# The indirect effects of cognitive function in the association between stroke and quality of life among older adults in a rural community: A cross-sectional study

**DOI:** 10.1007/s11136-026-04379-6

**Published:** 2026-08-29

**Authors:** Memory Lucy Mtambo, Tin Tin Su, Devi Mohan, Narelle Warren, Kia Fatt Quek

**Affiliations:** 1 Jeffrey Cheah School of Medicine and Health Sciences, Monash University Malaysia, Bandar Sunway, Selangor Malaysia; 2 School of Public Health, The University of Queensland, Brisbane, QLD Australia; 3 School of Social Sciences, Monash University, Melbourne, VIC Australia; 4 South East Asia Community Observatory (SEACO), Global Population Health, Jeffrey Cheah School of Medicine and Health Sciences, Monash University Malaysia, Bandar Sunway, Selangor Malaysia

**Keywords:** Cognitive function, Observational study, Low- and middle-income countries, Quality of life

## Abstract

**Purpose:**

We examined whether cognitive function statistically accounted for associations between stroke and quality of life (QOL).

**Methods:**

A cross-sectional survey included adults aged ≥ 60 years in Segamat, Malaysia. Cognition was assessed using the Identification and Intervention for Dementia in Elderly Africans instrument, and QOL was assessed using the WHOQOL-BREF. Stroke survivors were matched 1:3 with non-stroke participants by propensity scores based on age, sex, and education. Stroke status was the exposure, cognition the proposed statistical mediator, and four QOL domains, overall QOL, and overall health the outcomes. Models were adjusted for sociodemographic and vascular factors, accounted for matching and multiple testing, and *p* < 0.05 was considered statistically significant.

**Results:**

Among 596 participants, 149 reported stroke; the mean age was 70.36 years (SD 6.81); 41.6% female; 58.4% male. An indirect association between stroke and physical QOL via cognition was observed (β = −0.67, 95% CI − 1.30 to − 0.18; BH-adjusted *p* = 0.018). A residual correlation of ρ = 0.16 between cognition and physical-QOL models would attenuate this association to the null. The indirect association did not differ by sex (index β = −0.49, 95% bootstrap CI − 1.48 to 0.18; *p* = 0.164). Item-level physical-QOL analysis identified an indirect association with greater pain interference through lower cognition (β = −0.051, 95% CI − 0.113 to − 0.009; BH-adjusted *p* = 0.028).

**Conclusion:**

Reduced cognition partly accounted for associations of stroke with reduced physical QOL and greater pain interference. Findings were sensitive to modest unmeasured confounding but require confirmation from longitudinal studies.

**Supplementary Information:**

The online version contains supplementary material available at https://doi.org/10.1007/s11136-026-04379-6.

## Abstract (plain language summary)

This study examined whether difficulties with thinking, remembering, understanding, learning, and making decisions might explain why older adults with a history of stroke report a reduced satisfaction with their quality of life (QOL) compared to those without a stroke. We asked adults aged 60 years and above in Segamat, and each participant with a history of stroke was compared with three participants of a similar age, sex, and education who had not experienced a stroke. We used responses from 596 adults in this analysis, of whom 149 reported that they had previously been diagnosed with stroke by a doctor.

Our results suggested that difficulties with thinking, remembering, understanding, learning, and decision-making partly explained the relationship between stroke and reduced physical QOL. However, modest unmeasured differences in both thinking and remembering challenges and in physical QOL could have reduced this finding to zero. There was no evidence that this indirect relationship differed between males and females. The findings also suggested that thinking and remembering challenges partly explained the relationship between stroke and greater interference of pain with daily life.

These findings suggest that problems with thinking, remembering, understanding, learning, decision-making and pain are potential targets for community-based support services for stroke survivors. Studies that assess people before a stroke occurs and repeat assessments over time are needed to confirm exactly how these events influence one another.

**Supplementary Information:**

The online version contains supplementary material available at https://doi.org/10.1007/s11136-026-04379-6.

## Introduction

Stroke is a leading cause of disability in older adults worldwide, with an estimated 93.8 million people living with stroke and 160.5 million disability-adjusted life years (DALYs) attributable to stroke in 2021 [1, 2]. About 90% of stroke-related DALYs occur in low- and middle-income countries (LMICs) [1], yet limited evidence on stroke-related impairments comes from LMIC settings, which limits the applicability of support services to these settings [3]. Cognitive impairment is among the most disabling long-term consequences of stroke, affecting up to 60% of hospitalised survivors and nearly one-third of community-dwelling stroke populations [4, 5]. Reduced cognitive function is consistently associated with diminished quality of life (QOL) [6, 7], whereas stroke itself is independently associated with diminished QOL [8]. Stroke and cognitive deficits may influence QOL through distinct and overlapping pathways. Understanding these relationships is particularly important in rural communities, where access to professional support services may be limited, making tailored support difficult to obtain [9].

The 2010 Human Development Model–Disability Creation Process (HDM-DCP) conceptualises disability as arising from interactions between impairments and environmental factors that influence participation in life practice [10, 11]. Within this framework, stroke-related neurological dysfunction may impair cognitive functioning, limiting the capacity to perform daily activities, maintain meaningful social roles, and achieve functional independence [12–14]. These limitations may be influenced by sociodemographic characteristics, including age, sex, ethnicity, education, employment, and marital status, which affect cognitive reserve, socioeconomic circumstances, social participation, recovery, and available support [15–20]. Additionally, hypertension, diabetes, high cholesterol, heart disease, and smoking contribute to cerebrovascular pathology and may further exacerbate cognitive impairment and functional limitations [21]. Stroke, cognitive capacity, sociodemographic characteristics, and vascular risk factors may shape survivors' recovery and perceptions of QOL and require concurrent consideration for tailored interventions [22].

QOL reflects an individual's perception of their position in life within their cultural context and in relation to their goals, expectations, and concerns [23]. Its multidimensional and subjective nature makes understanding its determinants essential and aligns with the United Nations Sustainable Development Goals of promoting health and well-being (SDG 3) [24]. However, limited evidence has examined whether cognitive function statistically accounts for the association between stroke and QOL among older adults in rural LMIC communities. Hence, this study examined whether cognitive function statistically accounted for the association between stroke status and QOL among older adults living in a rural Malaysian community. We hypothesised that older adults with stroke would report lower QOL and that reduced cognitive function would statistically account for part of this association.

## Methods

We use the Strengthening the Reporting of Observational Studies in Epidemiology (STROBE) guidelines [25] and the Guideline for Reporting Mediation Analyses (AGReMA) [26] to report this study (Supplementary Tables S1-S2). We conducted a cross-sectional matched analysis of data from the South East Asia Community Observatory Health and Demographic Surveillance System (SEACO HDSS) collected in Segamat District, Johor, Malaysia. SEACO HDSS is a community-based surveillance platform established in 2011 [27], and this was its first Health Round survey to include objective cognitive assessment among adults aged ≥60 years. The Health Round survey was conducted between September 2023 and December 2024. Eligible participants had participated in the 2013 or 2018 SEACO rounds, provided informed consent, and could participate independently or with proxy assistance. Surveys completed entirely by proxy were excluded because cognitive screening required independent participation. Trained community-based multilingual fieldworkers conducted household interviews using electronic questionnaires, with identical procedures for all participants. Ethical approval was granted by the Monash University Human Research Ethics Committee (No. 38735). Written or video-recorded informed consent was obtained from all participants, and the survey adhered to the requirements of the Declaration of Helsinki [28].

The questionnaire assessed age, sex, ethnicity, marital status, education, current employment, hypertension, diabetes, high cholesterol, heart disease, and smoking history, with stroke status determined based on self-report of a previous stroke diagnosis. Cognitive function was assessed using the Identification and Intervention for Dementia in Elderly Africans (IDEA) screening instrument, with scores ranging from 0 to 15; higher scores indicate better cognition [29]. The IDEA has demonstrated excellent criterion validity in Malaysia [30]. QOL was assessed using the validated Malaysian 26-item WHOQOL-BREF, comprising physical, psychological, social relationships, and environmental domains, as well as single items assessing overall QOL and overall health [31]. Items were rated on a five-point Likert scale; negatively worded items 3, 4, and 26 were reverse-scored. Domain scores were calculated according to WHOQOL-BREF guidelines and transformed to a 0–100 scale, with higher scores indicating better QOL; overall QOL and overall health were analysed separately [32, 33].

This analysis was not prospectively registered, and data were obtained through the SEACO HDSS data-access processes [34]. The available sample comprised eligible SEACO participants with complete cognition scores and known stroke status, from whom the matched analytical sample was derived (Fig. 2). The data-specified primary analysis examined whether cognition statistically accounted for the association between stroke and six prespecified QOL outcomes using fully adjusted complete-case models. Unadjusted, multiply imputed, item-specific, and sex-stratified analyses were exploratory. Participants with stroke were matched 1:3 without replacement to those without stroke using nearest-neighbour propensity score matching (PSM) [35]. Propensity scores were estimated using continuous age, sex (male or female), and education (< 6 or ≥6 years), with exact matching on sex and education and a 0.2-standard-deviation calliper. Closest-distance ordering and the average treatment effect among the treated estimand were used. Covariate balance was assessed using absolute standardised mean differences (SMDs) (< 0.10), variance ratios close to 1, and empirical cumulative distribution function statistics close to 0 [36]. Variables not included in matching were addressed through regression adjustment, and matching variables were retained in the adjusted models as per Austin’s [37] recommendations.

**Fig. 1:**
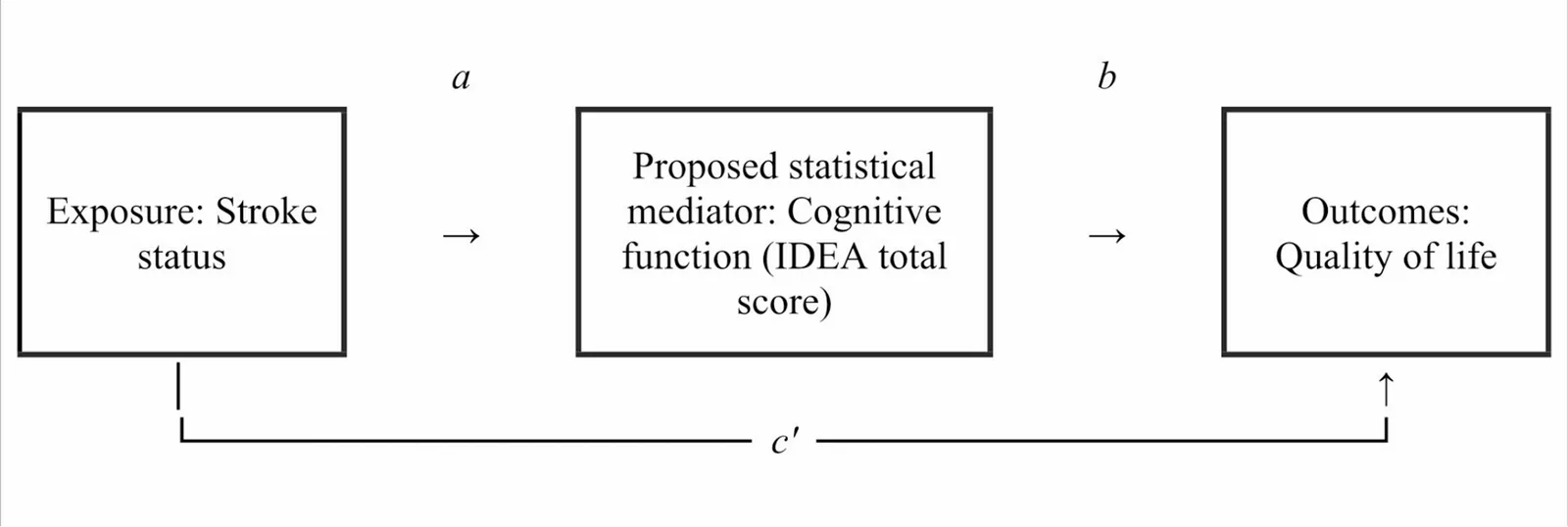
Analytical framework for assessing the indirect association between stroke status and quality of life through IDEA cognition score using cross-sectional data. Adapted from Hayes’ simple mediation model [38]. *Note* Path *a* represented the association between stroke status and IDEA cognition score. Path *b* represented the association between IDEA cognition score and QOL, conditional on stroke status and model covariates. The indirect association was calculated as *a* × *b*. The direct association (c′) represented the association between stroke status and QOL, conditional on IDEA cognition score and model covariates. The total association reflected the overall association between stroke status and QOL, without accounting for the IDEA cognition score. Alternative text: Path diagram showing stroke status as the exposure, IDEA cognition score as the statistical mediator, and quality of life as the outcome. Stroke status is linked to cognition via path a, cognition to quality of life via path b, and stroke status directly to quality of life via path c-prime

Participant characteristics were summarised using frequencies and percentages for categorical variables and means (standard deviations) and medians (interquartile ranges) for continuous variables. Groups were compared using Pearson’s chi-square or Fisher’s exact tests for categorical variables, and Welch’s *t*-tests or Mann–Whitney *U* tests for continuous variables, as appropriate. These were unadjusted descriptive comparisons and did not account for matched sets. Statistical indirect associations were estimated using a regression-based product-of-coefficients approach corresponding to Hayes’ simple mediation model (PROCESS Model 4) [38]. Stroke status was the exposure; the continuous IDEA cognition score was the proposed statistical mediator; and the four WHOQOL-BREF domains, overall QOL, and overall health were the outcomes (Fig. 1). The model was supported by evidence linking stroke with reduced cognition [39], cognition with QOL [7], and stroke with QOL [40]. Ordinary least-squares regression estimated the stroke–cognition association (path *a*) and cognition–QOL association conditional on stroke (path *b*); their product (*a* × *b*) represented the indirect association. Direct and total associations represented the stroke–QOL association conditional on cognition and without cognition in the outcome model, respectively, as illustrated in Fig. 1 [38]. Models assumed correct specification, linearity, independent residuals with constant variance, and no substantial multicollinearity. Causal interpretation would additionally require no unmeasured confounding of the stroke–cognition, cognition–QOL, and stroke–QOL associations [38, 41]. This cross-sectional design could not establish temporal ordering or exclude unmeasured confounding; hence, indirect effects were interpreted as statistical associations.

Dependence within matched sets was addressed by resampling each matched set with replacement in 2000 bootstrap samples, yielding 95% confidence intervals (CIs). Two-sided bootstrap *p*-values were twice the smaller proportion of valid estimates on either side of zero, capped at 1. The Benjamini–Hochberg (BH) correction was applied separately to the six QOL outcomes in both adjusted and unadjusted models [42]. Fully adjusted models included age, sex, ethnicity, marital status, education, current employment, hypertension, diabetes, high cholesterol, heart disease, and smoking history [15, 16]. Reference categories were no stroke, female sex, Malay ethnicity, not married, <6 years of education, not currently employed, absence of each vascular condition, and no smoking history. Age, IDEA, and QOL scores were continuous, and the analyses used outcome-specific complete cases. Sensitivity to unmeasured mediator–outcome confounding was assessed by varying the residual correlation (ρ) between mediator and outcome model errors and identifying the value at which the indirect association became zero [41].

Missingness was assessed using Little’s Missing Completely at Random (MCAR) test [43], and comparisons of participants with complete versus incomplete data. Multiple imputation by chained equations generated 20 datasets as a sensitivity analysis of the significant physical-QOL indirect association. Predictive mean matching was used for continuous QOL variables and logistic regression for binary variables; we combined estimates using Rubin’s rules and used cluster-robust standard errors to account for matched sets [44]. Additional exploratory analyses examined the seven physical-QOL items on their original five-point scales after reverse-scoring items 3 and 4. The primary product-of-coefficients and matched-set bootstrap procedures were used, with BH correction applied across the seven items. Item-specific indirect associations were interpreted separately and were not assumed to sum to the total physical QOL domain.

Sex was examined as an exploratory effect modifier of the physical-QOL indirect association because sex differences in QOL among stroke populations have been reported [45]. Sex was self-reported as male, female, or other; no participant selected “other”, and sex was conceptualised as biological characteristics [46]. The physical-QOL model included stroke status, cognition, sex, and a cognition-by-sex interaction. Conditional indirect associations were estimated for females and males, and their difference provided the index of conditional indirect association [38]. Two sensitivity models were examined: one excluded covariates that differed by sex, and the other adjusted only for hypertension, diabetes, and high cholesterol. CIs and *p*-values were obtained using the matched-set bootstrap procedure in the primary analysis.

Furthermore, post hoc Pearson correlations among WHOQOL-BREF outcomes were adjusted using the Holm–Bonferroni method [47], plus an average inter-domain correlation calculated separately by stroke status. Lastly, no a priori power calculation was conducted because this was a secondary analysis of an existing cohort. Simulation-based power analysis examined nine combinations of small (0.14), medium (0.39), and large (0.59) standardised *a* and *b* paths, following Fritz and MacKinnon [48]. Simulations maintained the 1:3 stroke-to-control ratio and used the primary bootstrap approach to estimate the minimum sample size for ≥80% power and expected power of the current sample. Analyses were conducted in R 4.5.1 using RStudio 2026.06.0+242 on macOS. PSM used MatchIt 4.7.2, mediator–outcome confounding sensitivity analyses used mediation 4.5.1, multiple imputation used mice 3.19.0, and matched-set bootstrapping used base R [49, 50]. Estimates were unstandardised and expressed in the original IDEA and QOL units; unless otherwise specified, two-sided *p*<0.05 indicated statistical significance.

## Results

**Fig. 2:**
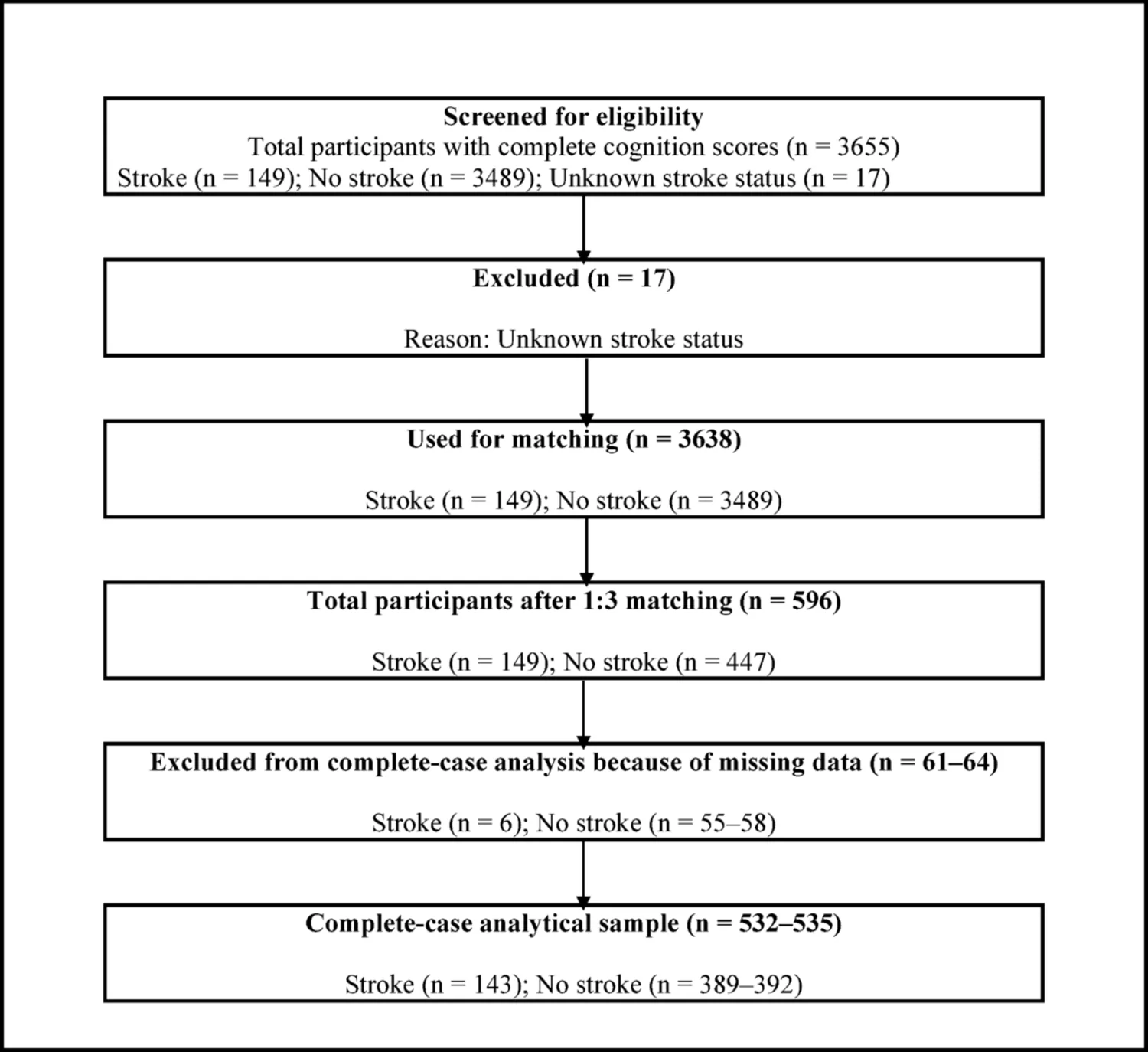
Flow diagram of participant selection. Alternative text: Flow diagram showing participant selection. Of 3655 participants with complete IDEA cognition scores, 17 with unknown stroke status were excluded, leaving 3638 participants for matching. After 1:3 matching, 596 participants were retained: 149 with stroke and 447 without stroke. Depending on the outcome, 61–64 participants were excluded for missing data, yielding complete-case analytical samples of 532–535 participants, including 143 with stroke and 389–392 without stroke. The sample size varies by QOL outcome because of outcome-specific missing data

Of the 3655 participants with complete IDEA cognition scores, 17 with unknown stroke status were excluded. After 1:3 PSM, 596 participants were included: 149 participants with stroke and 447 matched controls (Fig. 2). The mean age was 70.36 years (SD 6.81); 248 participants (41.6%) were female, and 348 (58.4%) were male (Table 1). Excellent balance was achieved for the matching variables: age, sex, and education, with all absolute SMDs below 0.10 (Supplementary Table S3).

**Table 1:** Entire matched sample participant characteristics and, by stroke status

Variable	Category	Total (n = 596)	No stroke (n = 447)	Stroke (n = 149)	*P* value
Age, years	Mean (SD)	70.36 (6.81)	70.35 (6.79)	70.38 (6.90)	0.956
	Median [IQR]	70.00 [65.00–74.00]	70.00 [65.00–74.00]	70.00 [65.00–74.00]	0.993
IDEA cognition score	Mean (SD)	12.35 (3.10)	12.55 (2.98)	11.74 (3.39)	0.010*
	Median [IQR]	13.00 [11.00–15.00]	13.00 [11.00–15.00]	13.00 [10.00–15.00]	0.004*
Sex	Female	248 (41.6%)	186 (41.6%)	62 (41.6%)	1.000
	Male	348 (58.4%)	261 (58.4%)	87 (58.4%)	
Ethnicity	Malay	482 (80.9%)	375 (83.9%)	107 (71.8%)	0.003*
	Chinese	68 (11.4%)	45 (10.1%)	23 (15.4%)	
	Indian/Other	46 (7.7%)	27 (6.0%)	19 (12.8%)	
Marital status	Not married	155 (26.0%)	120 (26.8%)	35 (23.5%)	0.419
	Married	441 (74.0%)	327 (73.2%)	114 (76.5%)	
Education	<6 years of education	148 (24.8%)	111 (24.8%)	37 (24.8%)	1.000
	≥6 years of education	448 (75.2%)	336 (75.2%)	112 (75.2%)	
Currently working	No	430 (72.3%)	311 (69.7%)	119 (79.9%)	0.017*
	Yes	165 (27.7%)	135 (30.3%)	30 (20.1%)	
Hypertension	No	196 (34.1%)	165 (38.6%)	31 (21.1%)	<0.001*
	Yes	378 (65.9%)	262 (61.4%)	116 (78.9%)	
Diabetes	No	367 (65.1%)	293 (70.1%)	74 (50.7%)	<0.001*
	Yes	197 (34.9%)	125 (29.9%)	72 (49.3%)	
High cholesterol	No	200 (36.5%)	169 (42.1%)	31 (21.1%)	<0.001*
	Yes	348 (63.5%)	232 (57.9%)	116 (78.9%)	
Heart disease	No	535 (90.1%)	404 (90.8%)	131 (87.9%)	0.311
	Yes	59 (9.9%)	41 (9.2%)	18 (12.1%)	
History of smoking	No	397 (66.6%)	307 (68.7%)	90 (60.4%)	0.064
	Yes	199 (33.4%)	140 (31.3%)	59 (39.6%)	

*Abbreviations*:IDEA, Identification and Intervention for Dementia in Elderly Africans; IQR, interquartile range; SD, standard deviation

Participant numbers (N) varied by variable because of missing data. Differences in participant characteristics between the stroke and non−stroke groups were assessed using Pearson's chi−square test or Fisher's exact test for categorical variables, and Welch's t−test or the Mann–Whitney U test for continuous variables, as appropriate. An asterisk (*) indicates p < 0.05

For the fully adjusted models, outcome-specific complete-case sample sizes ranged from 532 to 535 participants. Overall, 64 participants (10.7%) had incomplete data for at least one variable included in the fully adjusted analyses. Missingness was associated with some variables, such as being more common among non-stroke than among participants with stroke (58/447 [13.0%] vs 6/149 [4.0%]; *p* = 0.002). Little’s MCAR test was statistically significant, χ2 (187) = 281.81, *p* < 0.001, providing evidence against the MCAR assumption (Supplementary Tables S4–S6). All 149 original matched sets were represented in the complete-case analyses, and 142–143 sets (95.3%–96.0%) retained participants from both the stroke and control groups in fully adjusted models (Supplementary Table S7).

After adjustment for IDEA cognition score, age, sex, ethnicity, marital status, education, employment status, hypertension, diabetes, high cholesterol, heart disease and smoking history, stroke was associated with lower physical QOL (β = − 5.54; BH-adjusted *p* = 0.012), psychological QOL (β = − 2.64; BH-adjusted *p* = 0.039) and overall health (β = − 4.65; BH-adjusted *p* = 0.044). No statistically significant adjusted direct associations were observed for other QOL outcomes after BH correction. After adjustment for stroke status and the same sociodemographic and vascular covariates, higher IDEA cognition scores were associated with better physical QOL (β = 0.69; BH-adjusted *p* = 0.012), but not with the other QOL outcomes after BH correction. Moreover, in the fully adjusted complete-case model, a statistically significant indirect association between stroke and physical QOL via cognition was observed (β = − 0.67, 95% CI, − 1.30 to −0.18; BH-adjusted *p* = 0.018). The indirect association represented 10.8% of the total association. No statistically significant adjusted indirect associations were observed for other QOL outcomes after BH correction (Table 2).

**Table 2:** Unadjusted and adjusted direct and indirect associations of stroke with quality of life through cognition

Outcome	Model	N	Path a	Path a 95% CI	Path a BH adjusted p	Path b	Path b 95% CI	Path b BH adjusted p	Indirect effect	Indirect95% CI	Indirect BH adjusted p	Direct effect	Direct 95% CI	Direct BH adjusted p	Total effect	Total 95% CI	Total BH adjusted p
Physical QOL	Unadjusted	595	− 0.82	− 1.47 to − 0.23	0.011*	0.64	0.30 to 0.99	0.009*	− 0.52	− 1.08 to − 0.11	0.020*	− 7.99	− 10.51 to − 5.51	0.002*	− 8.51	− 11.03 to − 6.07	0.002*
Physical QOL	Adjusted	535	− 0.96	− 1.59 to − 0.31	0.006*	0.69	0.29 to 1.09	0.012*	− 0.67	− 1.30 to − 0.18	0.018*	− 5.54	− 8.28 to − 2.81	0.012*	− 6.21	− 9.10 to − 3.35	0.006*
Psychological QOL	Unadjusted	595	− 0.82	− 1.44 to − 0.24	0.011*	0.49	0.16 to 0.80	0.012*	− 0.4	− 0.92 to − 0.07	0.020*	− 4.01	− 6.40 to − 1.69	0.002*	− 4.41	− 6.80 to − 2.05	0.002*
Psychological QOL	Adjusted	535	− 0.96	− 1.58 to − 0.30	0.007*	0.29	− 0.07 to 0.67	0.199	− 0.28	− 0.75 to 0.06	0.205	− 2.64	− 4.95 to − 0.44	0.039*	-2.92	− 5.34 to − 0.65	0.022*
Social QOL	Unadjusted	591	− 0.83	− 1.51 to − 0.21	0.011*	0.27	− 0.03 to 0.54	0.124	− 0.22	− 0.60 to 0.04	0.139	− 0.72	− 2.73 to 1.24	0.491	− 0.95	− 2.90 to 0.99	0.364
Social QOL	Adjusted	532	− 0.96	− 1.59 to − 0.31	0.007*	0.19	− 0.14 to 0.50	0.253	− 0.19	− 0.54 to 0.14	0.255	0.4	− 1.74 to 2.38	0.699	0.21	− 1.96 to 2.13	0.825
Environmental QOL	Unadjusted	595	− 0.82	− 1.41 to − 0.25	0.011*	0.59	0.25 to 0.91	0.006*	− 0.48	− 0.95 to − 0.11	0.020*	− 1.5	− 3.49 to 0.54	0.168	− 1.98	− 3.95 to − 0.04	0.055
Environmental QOL	Adjusted	535	− 0.96	− 1.62 to − 0.29	0.006*	0.39	0.04 to 0.72	0.087	− 0.38	− 0.81 to − 0.03	0.093	− 0.5	− 2.53 to 1.55	0.699	− 0.88	− 2.82 to 1.09	0.446
Overall QOL	Unadjusted	596	− 0.81	− 1.43 to − 0.21	0.011*	0.27	− 0.18 to 0.72	0.294	− 0.21	− 0.67 to 0.17	0.301	− 4.09	− 7.55 to − 0.59	0.034*	− 4.31	− 7.72 to − 0.89	0.019*
Overall QOL	Adjusted	535	− 0.96	− 1.65 to − 0.35	0.006*	0.3	− 0.22 to 0.83	0.253	− 0.29	− 0.87 to 0.22	0.255	− 3.43	− 6.95 to 0.05	0.084	− 3.71	− 7.28 to − 0.26	0.061
Overall health	Unadjusted	596	− 0.81	− 1.41 to − 0.20	0.011*	0.22	− 0.28 to 0.68	0.369	− 0.18	− 0.70 to 0.23	0.377	− 5.92	− 9.73 to − 2.38	0.002*	− 6.1	− 9.84 to − 2.52	0.002*
Overall health	Adjusted	535	− 0.96	− 1.59 to − 0.33	0.007*	0.41	− 0.13 to 0.93	0.199	− 0.39	− 1.06 to 0.12	0.205	− 4.65	− 8.38 to − 0.82	0.044*	− 5.04	− 8.83 to − 1.11	0.022*

*Abbreviations*: BH, Benjamini–Hochberg; CI, confidence interval; IDEA, Identification and Intervention for Dementia in Elderly Africans; QOL, quality of life

Values are unstandardised estimates expressed in the original units of the IDEA cognition and QOL scores. Path a represents the association between stroke status and IDEA cognition score. Path b represents the association between IDEA cognition score and the QOL outcome, conditional on stroke status and the covariates included in the model. The indirect association (a × b) was calculated using the product−of−coefficients approach. The direct association represents the association between stroke status and the QOL outcome, conditional on IDEA cognition score and the model covariates. The total association represents the association between stroke status and the QOL outcome when the IDEA cognition score was excluded from the outcome model. In the unadjusted analysis, ordinary least squares regression was used to estimate path a, with stroke status as the predictor of the IDEA cognition score, and path b, with stroke status and the IDEA cognition score as predictors of the QOL outcome. Adjusted models additionally controlled for age, sex, ethnicity, marital status, education, current employment, hypertension, diabetes, high cholesterol, heart disease, and smoking history. Dependence within the 149 propensity−score−matched sets was addressed by resampling entire matched sets with replacement. The 95% confidence intervals are percentile intervals obtained from 2,000 matched−set bootstrap resamples. Raw two−sided bootstrap p−values were calculated directly from the empirical bootstrap distribution of each effect as twice the smaller proportion of bootstrap estimates on either side of zero (capped at 1). The Benjamini–Hochberg correction was then applied separately to the raw bootstrap p−values across the six QOL outcomes in the unadjusted and adjusted analyses; the BH−adjusted p−values are reported in the table. Outcome−specific complete−case analysis was used; therefore, sample sizes vary across QOL outcomes. An asterisk (*) indicates BH−adjusted p < 0.05

A separate model sensitivity analysis of the physical QOL showed that the estimated indirect association would be reduced to zero at a residual correlation of ρ = 0.16 between the cognition and physical-QOL models. This finding indicates sensitivity to modest unmeasured confounding of the cognition–physical QOL association (Supplementary Table S8). Moreover, in the exploratory analyses using seven separate models on their 5-point Likert scale, one for each of the physical QOL items, stroke was indirectly associated with greater pain interference through lower IDEA cognition scores in the fully adjusted pain-interference model (β = −0.051, 95% CI: −0.113 to −0.009; BH-adjusted p = 0.028) (Supplementary Table S9). No statistically significant adjusted indirect associations were observed for the other six physical QOL items after BH correction.

Multiple imputation was conducted as a sensitivity analysis for the physical QOL outcome. Participant characteristics and variable distributions were similar before and after imputation (Supplementary Table S10). In the sensitivity analysis using 20 imputed datasets (N = 596) and adjusting for the aforementioned covariates, the indirect association between stroke and physical QOL through cognition remained statistically significant (β = − 0.58, 95% CI −1.10 to −0.06; *p* = 0.030). The indirect association represented approximately 9.2% of the total association (95% CI 0.08%–18.34%) (Supplementary Table S11).

Moreover, an exploratory analysis examined whether the indirect association between stroke and physical QOL via cognition differed by sex. Variable comparisons between males and females are presented in Supplementary Table S12. In the fully adjusted model, there was no statistical evidence that the conditional indirect association between stroke and physical QOL through cognition differed by sex (β = −0.49, 95% bootstrap CI −1.48 to 0.18; p = 0.164). Descriptively, the conditional indirect association was statistically significant among males in the fully adjusted model (β = − 0.91, 95% bootstrap CI −1.90 to − 0.25; p = 0.003), but not among females (β = −0.41, 95% bootstrap CI −1.14 to 0.10; p = 0.114) (Table 3).

**Table 3:** Post hoc exploratory analysis of sex-specific indirect associations between stroke and physical quality of life through IDEA cognition: moderated-mediation and sensitivity analyses

Effect	Model	N	Beta (95% CI)	P value
Path a: Stroke -> IDEA cognition	Fully adjusted model	535	-0.96 (− 1.60 to − 0.35)	0.003*
Cognition -> physical QOL among females	Fully adjusted model	535	0.43 (− 0.11 to 0.99)	0.113
Cognition -> physical QOL among males	Fully adjusted model	535	0.94 (0.43 to 1.50)	<0.001*
Conditional indirect association among females	Fully adjusted model	535	-0.41 (− 1.14 to 0.10)	0.114
Conditional indirect association among males	Fully adjusted model	535	-0.91 (− 1.90 to − 0.25)	0.003*
Index of conditional indirect associations (males—females)	Fully adjusted model	535	-0.49 (− 1.48 to 0.18)	0.164
Path a: Stroke -> IDEA cognition	Excluding variables differing by sex: marital status, education, current employment, hypertension, diabetes, and history of smoking	546	-0.95 (− 1.57 to − 0.35)	0.001*
Cognition -> physical QOL among females	Excluding variables differing by sex: marital status, education, current employment, hypertension, diabetes, and history of smoking	546	0.29 (− 0.27 to 0.81)	0.304
Cognition -> physical QOL among males	Excluding variables differing by sex: marital status, education, current employment, hypertension, diabetes, and history of smoking	546	0.86 (0.35 to 1.40)	0.001*
Conditional indirect association among females	Excluding variables differing by sex: marital status, education, current employment, hypertension, diabetes, and history of smoking	546	-0.27 (− 0.93 to 0.25)	0.305
Conditional indirect association among males	Excluding variables differing by sex: marital status, education, current employment, hypertension, diabetes, and history of smoking	546	-0.81 (− 1.73 to − 0.18)	0.002*
Index of conditional indirect associations (males—females)	Excluding variables differing by sex: marital status, education, current employment, hypertension, diabetes, and history of smoking	546	-0.54 (− 1.48 to 0.12)	0.111
Path a: Stroke -> IDEA cognition	Adjusted for hypertension, diabetes and cholesterol only	537	-0.99 (− 1.64 to − 0.35)	0.003*
Cognition -> physical QOL among females	Adjusted for hypertension, diabetes and cholesterol only	537	0.45 (− 0.10 to 0.96)	0.112
Cognition -> physical QOL among males	Adjusted for hypertension, diabetes and cholesterol only	537	0.83 (0.36 to 1.38)	<0.001*
Conditional indirect association among females	Adjusted for hypertension, diabetes and cholesterol only	537	-0.44 (− 1.16 to 0.10)	0.115
Conditional indirect association among males	Adjusted for hypertension, diabetes and cholesterol only	537	-0.82 (− 1.75 to − 0.20)	0.003*
Index of conditional indirect associations (males—females)	Adjusted for hypertension, diabetes and cholesterol only	537	-0.37 (− 1.35 to 0.31)	0.253

Values are unstandardised estimates expressed in the original units of the IDEA cognition and physical QOL scores—CI, confidence interval; QOL, quality of life. Estimates were obtained using 2,000 matched−set bootstrap resamples, with percentile bootstrap confidence intervals (CIs) and two−sided bootstrap p−values. Two−sided bootstrap p−values were calculated directly from the empirical bootstrap distribution of each effect as twice the smaller proportion of bootstrap estimates on either side of zero (capped at 1). Consequently, p−values for the conditional indirect associations were derived from the bootstrap distributions of the products (path *a* × path *b*). They were not inherited from the corresponding path *b* estimates. Several p−values are numerically very similar to those of the corresponding path *b* coefficients; this reflects the empirical bootstrap distributions in these data rather than shared calculations. The conditional indirect association was calculated as the product of the stroke → IDEA cognition coefficient (path *a*) and the sex−specific coefficient for the association between IDEA cognition score and physical QOL (path *b*), conditional on stroke status and the covariates included in the model. The index of conditional indirect associations represents the difference between the conditional indirect associations for males and females (males minus females); a confidence interval containing zero indicates no evidence that the indirect association differed by sex. Higher IDEA cognition and physical QOL scores indicate better cognition and physical quality of life, respectively. Fully adjusted models controlled for age, ethnicity, marital status, education, current employment, hypertension, diabetes, high cholesterol, heart disease, and smoking history. The analysis was post hoc and exploratory. An asterisk (*) indicates *p* < 0.05

All 15 pairwise correlations among the six QOL outcomes were positive and statistically significant after Holm adjustment (adjusted p < 0.001) (Supplementary Table S13). Across the four WHOQOL-BREF domains, the mean of the six unique inter-domain correlations was identical among participants with stroke and matched controls (mean r = 0.43 in both groups) (Supplementary Table S14). Lastly, simulation-based power analysis using the analytical methods implemented in this study indicated limited power in the current sample when path b was small: 59.6% for the small–small, 68.0% for the medium–small, and 60.0% for the large–small scenario (Supplementary Table S16). Simulations suggested that a total sample of 800–1,100 participants would be required to achieve at least 80% power when path b was small (Supplementary Table S15).

## Discussion

In this matched cross-sectional sample of older adults from a rural LMIC setting, cognitive function partially accounted for the association between stroke and physical QOL, with no evidence that the indirect association differed by sex. These findings suggest that cognitive function may represent one pathway through which stroke is associated with reduced physical QOL. The indirect association was statistically significant among males but not among females, and is descriptive only. Moreover, sensitivity analysis indicated that the observed indirect association was sensitive to relatively small amounts of unmeasured confounding between cognition and physical QOL, warranting caution in interpreting the results. Nevertheless, few studies have examined cognitive function as a potential intermediate factor linking stroke and QOL, making these findings a valuable contribution to the literature and adding to the evidence for achieving SDG 3 [24].

Moreover, stroke was independently associated with substantially lower physical QOL, psychological QOL, and overall health, aligning with prior QOL work in stroke populations [8, 51, 52]. Hence, interdisciplinary stroke care should aim to assess all items in these outcomes to support personalised interventions. No statistically significant adjusted direct associations were observed for the remaining QOL outcomes after BH correction. However, the findings indicate moderate correlations among all QOL domains, as observed in other adult population studies [53]. Hence, stroke and older-adult interventions should concurrently target all domains to achieve comprehensive improvements in QOL. Notably, cognitive function showed significant independent associations with physical QOL, consistent with prior evidence [22, 54]. However, the cross-sectional design does not establish that maintaining cognition would improve physical QOL and requires longitudinal studies.

Furthermore, separate models of each of the seven physical QOL items showed that stroke was associated with greater pain interference through lower cognition, a finding reported in another study [55, 56]. From an HDM-DCP perspective, reduced cognitive function among stroke survivors may be one pathway statistically linking stroke to greater pain interference and overall reduction in physical QOL [11]. These findings support further evaluation of combined cognitive screening and pain assessment in community-based stroke care. However, limited power under several small-path scenarios means that non-significant indirect associations for other QOL outcomes should not be interpreted as evidence of no association. Lastly, Table 4 extends hypothesis-generating recommendations derived from the findings across the three spatial scales of micro-, meso-, and macro-levels of influences for community-based interventions [57]. Although presented as distinct, these scales are interrelated and overlapping, reflecting shared responsibilities across individuals, healthcare systems, and broader policy environments [58].

**Table 4:** Hypothesis generating recommendations for actions across different spatial scales based on findings for improving QOL among older adults with stroke in rural settings

Micro level: Individuals, families, and carer involvement (Individual & family-based actions)	Meso level: Healthcare and support services: acute and long-term medical and rehabilitation services (Service delivery & community continuity)	Macro level: Society and policy environment (systems, policy & population-level actions)
Encourage home-based cognitive stimulating activities tailored to local practices	Integrate cognitive and QOL screening into routine stroke care in community-based services	Embed cognitive care within rural healthy ageing and stroke strategies, linked to community-based services
Support pain self-management practices, such as activity pacing, appropriate exercise, relaxation techniques, comfortable positioning, and the safe use of prescribed and over the counter pain medication	Use screening results to tailor rehabilitation, prioritising pain interference within physical QOL	Invest in primary care capacity to manage stroke and cognition alongside coexisting pain and other vascular conditions
Promote participation in community screening for vascular risk, cognition, and QOL where available	Offer diverse pain management options tailored to individual needs	

## Limitations of the study

First, the cross-sectional design precludes causal or temporal inference, and reverse causation is possible. Secondly, a residual mediator–outcome confounding also remains possible; a residual correlation of ρ = 0.16 between the cognition and physical-QOL model errors would attenuate the primary indirect association to the null. Thirdly, stroke history was self-reported, without information on stroke date, type, or severity. Apart from that, the community-based sample may be affected by survival and selection biases because hospitalised individuals and those unable to complete cognitive screening independently were excluded. Stroke-related motor or visual impairments, aphasia, and neglect may also have influenced cognitive scores. Moreover, although multiple imputation addresses missing data, imputed values may not reflect actual responses, while complete-case analyses may be biased. Additionally, simulation-based power analysis indicated limited power in some small-path scenarios; thus, non-significant indirect associations should be interpreted with caution. Furthermore, IDEA is a screening tool and cannot diagnose cognitive impairment, which requires a comprehensive clinical assessment. Evidence also indicates that subjective health ratings may be higher in rural and developing countries than in urban and developed countries, despite weaker objective health indicators [59]. Moreover, generalisability is limited to community-dwelling adults aged ≥60 years from one rural Malaysian district. Lastly, sociocultural and contextual factors, including ageing expectations, substantially shape perceived QOL [23]. Hence, participant-reported QOL should be interpreted in the context of objective measures of disability, risk, activity, and participation for effective management. Despite these limitations, a key strength of this research is the use of a community-based sample from a rural LMIC setting, where population-level evidence on stroke, cognition and QOL is limited. Further longitudinal research is needed to examine how cognitive function evolves and how these changes influence QOL trajectories among stroke survivors and older adults in rural communities.

## Conclusion

This rural Malaysian cross-sectional study found that reduced cognitive function statistically accounted for part of the associations of stroke with lower physical QOL and greater pain interference. However, the cross-sectional design precludes temporal or causal inference, and the indirect association between physical QOL and the outcome was sensitive to modest unmeasured mediator–outcome confounding. There was no evidence that the indirect association differed by sex. These findings identify cognitive challenges and pain interference as priorities for further research in longitudinal studies.

## Electronic Supplementary Material

Below is the link to the electronic supplementary material.

Supplementary file1 (DOCX 96 KB)

## Data Availability

The data that support the findings of this study are available from the SEACO HDSS, Monash University Malaysia, subject to approval through the SEACO data access process. Information on data access is available at https://www.monash.edu.my/seaco.

## Declarations

### Patient, public or community engagement

SEACO engages the public and community throughout all stages of research conducted in the community, from planning and implementation to dissemination and policy engagement. Details on how SEACO engages the public and community can be found at https://www.monash.edu.my/seaco/public-and-community-involvement-and-engagement.

### Use of generative artificial intelligence tools

Grammarly for Education, provided through Monash University Malaysia, was used to support language refinement during manuscript preparation. All outputs were reviewed and edited by the authors, who take full responsibility for the final submitted content.
