# Supplementary material for: The indirect effects of cognitive function in the association between stroke and quality of life among older adults in a rural community: A cross-sectional study
Source: Qual Life Res. 2026 Aug 29;35(10):274. doi: 10.1007/s11136-026-04379-6 (PMC13525938; doi:10.1007/s11136-026-04379-6)
Supplement: Supplementary file 1 [file 11136_2026_4379_MOESM1_ESM.docx]

**The indirect effects of cognitive function in the association between stroke and quality of life among older adults in a rural community: A cross-sectional study**

**The indirect effects of cognitive function in the association between stroke and quality of life among older adults in a rural community: A cross-sectional study**

Memory Lucy Mtambo,^a^, Tin Tin Su,^a,d^, Devi Mohan,^a,b^, Narelle Warren,^c^, Kia Fatt Quek,^a^

**The indirect effects of cognitive function in the association between stroke and quality of life among older adults in a rural community: A cross-sectional study**

Memory Lucy Mtambo, BSc, MSc^a^, Tin Tin Su, MBBS, MSc, CHHM, PhD^a,d^, Devi Mohan, MBBS, MD, GCHE, PhD^a,b^, Narelle Warren, PhD^c^, Kia Fatt Quek, MAppStat, MPhil, PhD^a^

**Author affiliations**

^a^ Jeffrey Cheah School of Medicine and Health Sciences, Monash University Malaysia, Bandar Sunway, Selangor, Malaysia

^b^ School of Public Health, The University of Queensland, Brisbane, Queensland,

Australia

^c^ School of Social Sciences, Monash University, Melbourne, Victoria, Australia

^d^ South East Asia Community Observatory (SEACO), Global Population Health, Jeffrey Cheah School of Medicine and Health Sciences, Monash University Malaysia, Bandar Sunway, Selangor, Malaysia,

**Notes:** Participant numbers (N) differed across analyses because of missing data. Missingness ranged from 1 to 48 participants per variable. In the fully adjusted complete-case analyses, 61–64 participants were excluded (6 participants with stroke and 55–58 without stroke), resulting in analytical samples of 532–535 participants (143 participants with stroke and 389–392 without stroke). Values are unstandardised estimates expressed in the original units of the IDEA cognition and QOL scores. Unless otherwise specified, statistical significance was defined as a two-sided p < 0.05. Estimates were obtained using 2,000 matched-set bootstrap resamples, with percentile bootstrap confidence intervals (CIs) and two-sided bootstrap p-values. Welch's t-test was used to compare the means of continuous variables between two groups. Mann–Whitney U tests were used to compare continuous variables that were not normally distributed. Pearson's chi-square test was used to compare categorical variables when expected cell counts were adequate, and Fisher's exact test was used when any expected cell count was <5. Absolute standardised mean difference (SMD) values <0.10 indicate acceptable covariate balance. Variance ratios are presented for continuous variables only and are therefore reported as not applicable (NA) for binary variables. An asterisk (*) indicates statistical significance.

**Abbreviations:** AGReMA, A Guideline for Reporting Mediation Analyses; BH, Benjamini–Hochberg; CI, confidence interval; eCDF, empirical cumulative distribution function; IDEA, Identification and Intervention for Dementia in Elderly Africans; IQR, interquartile range; MI, multiple imputation; PSM, propensity score matching QOL, quality of life; SD, standard deviation; SMD, standardised mean difference; STROBE, Strengthening the Reporting of Observational Studies in Epidemiology; WHO, World Health Organization

**Additional information on statistical methods**

**Data analysis software and packages**

All analyses were conducted in R version 4.5.1 (R Foundation for Statistical Computing, Vienna, Austria) using RStudio version 2026.06.0+242 (Posit Software, PBC, Boston, MA, USA) on macOS Tahoe 26.6. PSM was performed using the MatchIt package (version 4.7.2), sensitivity analyses for unmeasured statistical mediator–outcome confounding were conducted using the mediation package (version 4.5.1), and multiple imputation was performed using the mice package (version 3.19.0). The matched-set bootstrap procedure was implemented using base R functions

**Simulation-based power analysis**

A simulation-based power analysis was conducted to estimate the sample size required to detect indirect associations under nine combinations of assumed standardised path coefficients in future studies. Standardised path coefficients of 0.14, 0.39, and 0.59, representing small, medium, and large effects, respectively, were selected from the mediation simulation scenarios described by Fritz and MacKinnon **[1].** For each scenario, datasets were simulated across a range of sample sizes while maintaining a 1:3 stroke-to-control ratio. The analysis was based on general assumed combinations of standardised path coefficients rather than effect-size estimates from previous stroke, cognition, or QOL studies, as suitable prior estimates were unavailable. Indirect associations were estimated using the same product-of-coefficients bootstrap procedure as in the primary analysis, and the minimum sample size required to achieve at least 80% power was identified for each effect-size scenario. Finally, the same simulation framework was applied to estimate the expected statistical power of the current study sample under each assumed effect-size scenario

**Handling of missing data**

Missing data were observed, particularly for vascular variables, as participants reported never having undergone these tests. The overall pattern of missing data was assessed using Little’s test of Missing Completely at Random (MCAR) [2]. The null hypothesis was that the data were MCAR; a statistically significant result was interpreted as evidence against the MCAR assumption. Additional analyses compared participants with complete data and those with any missing data on observed characteristics to assess whether missingness was associated with observed variables. Associations between missingness and observed characteristics were considered suggestive of a Missing at Random (MAR) mechanism. Missing data were handled using multiple imputation by chained equations with 20 completed datasets. A sensitivity analysis repeated the mediation analysis using the multiply imputed dataset to assess the impact of complete-case analysis on the statistically significant indirect association. Predictive mean matching was used for continuous QOL variables, and logistic regression was used for binary variables. Analyses were performed separately for each dataset and combined using Rubin’s rules, with cluster-robust standard errors to account for matched sets

**Sex-exploratory model**

The mediator model estimated the association between stroke status and IDEA cognition score (path a). The physical-QOL outcome model included stroke status, IDEA cognition score, sex, and an IDEA cognition-by-sex interaction term. This allowed the association between cognition and physical QOL (path b) to differ between females and males. Conditional indirect associations were calculated separately for females and males as the product of the stroke–cognition coefficient and the corresponding sex-specific cognition–physical-QOL coefficient. The index of conditional indirect association was calculated as the difference between the male and female conditional indirect associations, providing a formal test of whether the indirect association differed by sex [38]. **Two sensitivity models were also examined: one excluding covariates that differed between females and males and another adjusting only for hypertension, diabetes, and high cholesterol.** CIs were calculated using the same matched-set bootstrap procedure as in the primary analysis. Two-sided bootstrap p-values were calculated separately for each reported effect from its own bootstrap distribution, including the sex-specific path b coefficients

**Supplementary Table S1: STROBE Statement; Checklist of items that should be included in reports of cross-sectional studies**

| **Item** | **Item No.** | **Recommendation** | **Location in the manuscript** |
| --- | --- | --- | --- |
| **Title and abstract** | | | |
| Title and abstract | 1(a) | Indicate the study's design with a commonly used term in the title or the abstract. | Page 1 |
|  | 1(b) | Provide in the abstract an informative and balanced summary of what was done and what was found. | Pages 2–3 |
| **Introduction** | | | |
| Background/rationale | 2 | Explain the scientific background and rationale for the investigation being reported. | Pages 5–6 |
| Objectives | 3 | State specific objectives, including any prespecified hypotheses. | Page 6 |
| **Methods** | | | |
| Study design | 4 | Present key elements of the study design early in the paper. | Page 6 |
| Setting | 5 | Describe the setting, locations, and relevant dates, including periods of recruitment, exposure, follow-up, and data collection. | Page 6 |
| Participants | 6(a) | Give the eligibility criteria, and the sources and methods of selection of participants. | Pages 6–8; Figure 2 (page 15) |
| Variables | 7 | Clearly define all outcomes, exposures, predictors, potential confounders, and effect modifiers. Give diagnostic criteria, if applicable. | Pages 7–10; Figure 1 (page 14) |
| Data sources/measurement | 8* | For each variable of interest, give sources of data and details of methods of assessment (measurement). Describe the comparability of assessment methods if there is more than one group. | Pages 6–7 |
| Bias | 9 | Describe any efforts to address potential sources of bias. | Pages 8–10 and 23–25; Supplementary Tables S3–S8 |
| Study size | 10 | Explain how the study size was arrived at. | Pages 7 and 10–11; Figure 2 (page 15); Supplementary Tables S15–S16 |
| Quantitative variables | 11 | Explain how quantitative variables were handled in the analyses. If applicable, describe which groupings were chosen and why. | Pages 7–11; Table 1 (pages 16–17) |
| Statistical methods | 12(a) | Describe all statistical methods, including those used to control for confounding. | Pages 8–11 |
|  | 12(b) | Describe any methods used to examine subgroups and interactions. | Page 10 |
|  | 12(c) | Explain how missing data were addressed. | Pages 9–10; Supplementary File pages 1–2 and Supplementary Tables S4–S7 |
|  | 12(d) | If applicable, describe analytical methods taking account of the sampling strategy. | Pages 8–9 (matching and matched-set resampling) |
|  | 12(e) | Describe any sensitivity analyses. | Pages 9–10 |
| **Results** | | | |
| Participants | 13(a)* | Report numbers of individuals at each stage of study - e.g., numbers potentially eligible, examined for eligibility, confirmed eligible, included in the survey, completing follow-up, and analysed. | Page 11; Figure 2 (page 15) |
|  | 13(b)* | Give reasons for non-participation at each stage. | Figure 2 (page 15) |
|  | 13(c)* | Consider use of a flow diagram. | Figure 2 (page 15) |
| Descriptive data | 14(a)* | Give characteristics of study participants (e.g., demographic, clinical, social) and information on exposures and potential confounders. | Page 11; Table 1 (pages 16–17) |
|  | 14(b)* | Indicate the number of participants with missing data for each variable of interest. | Page 11; Table 1 footnote (page 17); Supplementary Tables S4–S7 |
| Outcome data | 15* | Report numbers of outcome events or summary measures. | Pages 12–13; Table 2 (pages 18–19) |
| Main results | 16(a) | Give unadjusted estimates and, if applicable, confounder-adjusted estimates and their precision (e.g., 95% confidence interval). Make clear which confounders were adjusted for and why they were included. | Pages 5–6, 8–9, and 12; Table 2 (pages 18–19) |
|  | 16(b) | Report category boundaries when continuous variables were categorised. | Page 8; Table 1 (pages 16–17; education: <6 vs ≥6 years) |
|  | 16(c) | If relevant, consider translating estimates of relative risk into absolute risk for a meaningful time period. | Not applicable - continuous QOL outcomes were analysed; no relative risks were estimated |
| Other analyses | 17 | Report other analyses done - e.g., analyses of subgroups and interactions, and sensitivity analyses. | Pages 12–13; Table 3 (pages 20–21); Supplementary Tables S8–S16 |
| **Discussion** | | | |
| Key results | 18 | Summarise key results with reference to study objectives. | Page 23 |
| Limitations | 19 | Discuss limitations of the study, taking into account sources of potential bias or imprecision. Discuss both the direction and magnitude of any potential bias. | Pages 23–25; sensitivity estimate on page 12 and Supplementary Table S8 |
| Interpretation | 20 | Give a cautious overall interpretation of results, considering objectives, limitations, multiplicity of analyses, results from similar studies, and other relevant evidence. | Pages 23–25 |
| Generalisability | 21 | Discuss the generalisability (external validity) of the study results. | Pages 24–25 |
| **Other information** | | | |
| Funding | 22 | Give the source of funding and the role of the funders for the present study and, if applicable, for the original research on which the present article is based. | Page 26 |

*Give information separately for exposed and unexposed groups.

**Supplementary Table S2: A Guideline for Reporting Mediation Analyses (AGReMA); 25-item- Long-Form Checklist**

| **Section and topic** | **Item No.** | **Item description** | **Location in the manuscript** |
| --- | --- | --- | --- |
| Title | 1 | Identify that the study uses mediation analyses | Page 1 |
| Abstract | 2 | Provide a structured summary of the objectives, methods, results, and conclusions specific to mediation analyses | Pages 2–3 |
| Background and rationale | 3 | Describe the study background and theoretical rationale; include rationale for exposure→mediator and mediator→outcome relationships | Pages 5–6 and 8 |
| Objectives | 4 | State objectives specific to mechanisms of interest | Page 6 |
| Study registration | 5 | Provide protocol/study registration if applicable | Page 7 (not prospectively registered) |
| Study design and source of data | 6 | Specify study design and data source | Pages 6–7 |
| Participants | 7 | Describe target population, eligibility, locations and dates | Pages 6–7 |
| Sample size | 8 | State whether a sample size calculation was conducted for mediation analyses | Pages 10–11 (no a priori calculation and simulation methods); page 13; Supplementary Tables S15–S16 |
| Effects of interest | 9 | Specify the effects of interest | Pages 8–9; Figure 1 (page 14) |
| Assumed causal model | 10 | Include a graphic representation of the assumed causal model | Pages 8–9; Figure 1 (page 14) |
| Causal assumptions | 11 | Specify assumptions about the causal model | Page 9 |
| Measurement | 12 | Describe exposures, mediators, outcomes, confounders, moderators and measurement | Pages 7–10; Figure 1 (page 14) |
| Measurement levels | 13 | Describe measurement levels if relevant | Pages 7–10 |
| Statistical methods | 14 | Describe statistical methods, software and package used | Pages 8–11 |
| Sensitivity analyses | 15 | Describe sensitivity analyses used | Pages 9–10 |
| Ethical approval | 16 | Name ethics committee and consent procedures | Pages 7 and 27 |
| Participants (Results) | 17 | Describe baseline characteristics and participant flow | Page 11; Figure 2 (page 15); Table 1 (pages 16–17) |
| Outcomes and estimates | 18 | Report point estimates and uncertainty | Pages 12–13; Table 2 (pages 18–19); Table 3 (pages 20–21) |
| Sensitivity parameters | 19 | Report sensitivity analysis results | Pages 12–13; Table 3 (pages 20–21); Supplementary Tables S8 and S10–S11 |
| Limitations | 20 | Discuss study limitations and potential bias | Pages 24–25 |
| Interpretation | 21 | Interpret findings considering assumptions, uncertainty and generalisability | Pages 23–25 |
| Implications | 22 | Discuss implications for practice, policy and science | Table 4 (page 22); pages 23–25 |
| Funding and role of sponsor | 23 | List funding sources and sponsor role | Page 26 |
| Conflicts of interest | 24 | State conflicts of interest and financial disclosures | Page 27 |
| Data and code | 25 | Provide statement on data/code availability (encouraged) | Page 28 (data access statement) |

# **Supplementary Table S3: Covariate balance used for matching before and after propensity score matching**

| Covariate | Before Matching | After Matching | Balance Diagnostics After Matching | | |
| --- | --- | --- | --- | --- | --- |
|  | SMD | SMD | Variance Ratio | eCDF Mean | eCDF Max |
| Age | 0.218 | 0.005 | 1.031 | 0.001 | 0.007 |
| Sex | 0.359 | 0.000 | NA | 0.000 | 0.000 |
| Education | 0.022 | 0.000 | NA | 0.000 | 0.000 |

**^Notes:^** ^Absolute standardised mean difference (SMD) values <0.10 indicate acceptable covariate balance. Variance ratios are presented for continuous variables only and are therefore reported as not applicable (NA) for binary variables. Before matching, age (SMD = 0.218) and sex (SMD = 0.359) showed imbalance between participants with and without stroke, whereas education was already well balanced (SMD = 0.022). After matching, all matching covariates achieved excellent balance (all absolute SMDs <0.10). The variance ratio for age was 1.031, and the empirical cumulative distribution function (eCDF) mean and maximum statistics were close to zero, indicating successful covariate balance^.

**Matching procedure**

 Propensity scores were estimated using logistic regression with stroke status as the dependent variable and age (continuous), sex (female or male), and education (<6 vs ≥6 years) as predictors. Stroke participants were matched to non-stroke participants using 1:3 nearest-neighbour propensity-score matching without replacement, targeting the average treatment effect among the treated (ATT). Exact matching was required for sex and education, whereas age was balanced using propensity scores with a calliper of 0.2 standard deviations of the logit of the propensity score. Matches were selected using closest-distance ordering (m.order = "closest"). Matching was performed in R version 4.5.1 using the MatchIt package (version 4.7.2). The final matched sample comprised 149 stroke participants and 447 matched controls (149 matched sets). Remaining baseline differences in variables not used for matching were accounted for through covariate adjustment in the regression analyses.

^.^

**Supplementary Table S4: Summary of missing data in the propensity-score-matched sample (N = 596)**

| **Measure** | **Overall** | **No stroke** | **Stroke** | **P value** | **Test** |
| --- | --- | --- | --- | --- | --- |
| Variables assessed | 18 |  |  |  |  |
| Variables with at least one missing value | 9 of 18 (50.0%) |  |  |  |  |
| Total missing data cells | 113 of 10728 (1.05%) |  |  |  |  |
| Participants with at least one missing value | 64 of 596 (10.7%) | 58 of 447 (13.0%) | 6 of 149 (4.0%) | 0.002* | Pearson chi-square test |

| **Assessment of the missing-data mechanism using Little’s Missing Completely at Random test** | |
| --- | --- |
| Purpose | To assess whether the overall pattern of missing data was Missing Completely at Random (MCAR). |
| Null hypothesis | Missing values are Missing Completely at Random (MCAR). |
| Alternative hypothesis | Missing values are not Missing Completely at Random. |
| Variables analysed | IDEA cognition score; age; sex; ethnicity; marital status; education; current employment status; hypertension; diabetes; high cholesterol; heart disease; history of smoking; physical, psychological, social, and environmental QOL; overall QOL; and overall health |
| Statistical test | Little's MCAR test. |
| Result | Little's MCAR test was statistically significant (χ² = 281.81, df = 187, p <0.001), indicating that the missing data were not meeting MCAR assumptions |

**Supplementary Table S5: Comparison of participant characteristics between complete cases (n = 532) and participants with any missing data (n = 64); N = 596**

| **Variable** | **Category or statistic** | **Complete (n = 532)** | **Missing (n = 64)** | **Test** | **P value** |
| --- | --- | --- | --- | --- | --- |
| Age (years) | Mean (SD) | 70.17 (6.78) | 71.89 (6.99) | Welch's t-test | 0.066 |
|  | Median (Q1, Q3) | 70.00 (65.00, 74.00) | 71.00 (65.75, 77.00) | Mann–Whitney U test | 0.062 |
| IDEA cognitive score | Mean (SD) | 12.45 (3.04) | 11.48 (3.49) | Welch's t-test | 0.037* |
|  | Median (Q1, Q3) | 13.00 (11.00, 15.00) | 12.00 (9.50, 15.00) | Mann–Whitney U test | 0.029* |
| Stroke status | No stroke | 389 (73.1%) | 58 (90.6%) | Pearson chi-square test | 0.002* |
|  | Stroke | 143 (26.9%) | 6 (9.4%) |  |  |
| Sex | Female | 227 (42.7%) | 21 (32.8%) | Pearson chi-square test | 0.131 |
|  | Male | 305 (57.3%) | 43 (67.2%) |  |  |
| Ethnicity | Malay | 430 (80.8%) | 52 (81.2%) | Fisher's exact test | 0.878 |
|  | Chinese | 60 (11.3%) | 8 (12.5%) |  |  |
|  | Indian/Other | 42 (7.9%) | 4 (6.2%) |  |  |
| Marital status | Not married | 132 (24.8%) | 23 (35.9%) | Pearson chi-square test | 0.055 |
|  | Married | 400 (75.2%) | 41 (64.1%) |  |  |
| Education | <6 years | 127 (23.9%) | 21 (32.8%) | Pearson chi-square test | 0.118 |
|  | ≥6 years | 405 (76.1%) | 43 (67.2%) |  |  |
| Employment status | Currently not working | 386 (72.6%) | 44 (69.8%) | Pearson chi-square test | 0.649 |
|  | Currently working | 146 (27.4%) | 19 (30.2%) |  |  |
| Hypertension | No hypertension | 174 (32.7%) | 22 (52.4%) | Pearson chi-square test | 0.010* |
|  | Hypertension | 358 (67.3%) | 20 (47.6%) |  |  |
| Diabetes | No diabetes | 340 (63.9%) | 27 (84.4%) | Pearson chi-square test | 0.018* |
|  | Diabetes | 192 (36.1%) | 5 (15.6%) |  |  |
| High cholesterol | No high cholesterol | 193 (36.3%) | 7 (43.8%) | Pearson chi-square test | 0.541 |
|  | High cholesterol | 339 (63.7%) | 9 (56.2%) |  |  |
| Heart disease | No heart disease | 479 (90.0%) | 56 (90.3%) | Pearson chi-square test | 0.943 |
|  | Heart disease | 53 (10.0%) | 6 (9.7%) |  |  |
| History of smoking | No history of smoking | 356 (66.9%) | 41 (64.1%) | Pearson chi-square test | 0.647 |
|  | History of smoking | 176 (33.1%) | 23 (35.9%) |  |  |

^Welch’s t-test was used to compare the means of continuous variables between two groups.^

^Mann–Whitney U test was used to compare the distributions or medians of continuous variables that were not normally distributed.^

^Pearson’s chi-square test was used to compare categorical variables when expected cell counts were adequate.^

^Fisher's exact test was used for categorical variables when expected cell counts were small (i.e., <5).^ **^An asterisk (*) indicates statistical significance at p < 0.05^**

**Supplementary Table S6. Detailed missing data, overall and by stroke status (N = 596)**

| **Variable** | **Total missing (n = 596)** | **No stroke missing (n = 447)** | **Stroke missing (n = 149)** | **P value** | **Test** |
| --- | --- | --- | --- | --- | --- |
| IDEA cognition score | 0 (0.0%) | 0 (0.0%) | 0 (0.0%) |  |  |
| Age, years | 0 (0.0%) | 0 (0.0%) | 0 (0.0%) |  |  |
| Sex | 0 (0.0%) | 0 (0.0%) | 0 (0.0%) |  |  |
| Ethnicity | 0 (0.0%) | 0 (0.0%) | 0 (0.0%) |  |  |
| Marital status | 0 (0.0%) | 0 (0.0%) | 0 (0.0%) |  |  |
| Education | 0 (0.0%) | 0 (0.0%) | 0 (0.0%) |  |  |
| Currently working | 1 (0.2%) | 1 (0.2%) | 0 (0.0%) | 1.000 | Fisher's exact test |
| Hypertension | 22 (3.7%) | 20 (4.5%) | 2 (1.3%) | 0.079 | Pearson chi-square test |
| Diabetes | 32 (5.4%) | 29 (6.5%) | 3 (2.0%) | 0.036* | Pearson chi-square test |
| High cholesterol | 48 (8.1%) | 46 (10.3%) | 2 (1.3%) | <0.001* | Pearson chi-square test |
| Heart disease | 2 (0.3%) | 2 (0.4%) | 0 (0.0%) | 1.000 | Fisher's exact test |
| History of smoking | 0 (0.0%) | 0 (0.0%) | 0 (0.0%) |  |  |
| Physical QOL | 1 (0.2%) | 1 (0.2%) | 0 (0.0%) | 1.000 | Fisher's exact test |
| Psychological QOL | 1 (0.2%) | 1 (0.2%) | 0 (0.0%) | 1.000 | Fisher's exact test |
| Social QOL | 5 (0.8%) | 5 (1.1%) | 0 (0.0%) | 0.339 | Fisher's exact test |
| Environmental QOL | 1 (0.2%) | 1 (0.2%) | 0 (0.0%) | 1.000 | Fisher's exact test |
| Overall QOL | 0 (0.0%) | 0 (0.0%) | 0 (0.0%) |  |  |
| Overall health | 0 (0.0%) | 0 (0.0%) | 0 (0.0%) |  |  |

An asterisk (*) indicates statistical significance at p < 0.05

**Supplementary Table S7: Complete-case participant numbers and retention of matched sets across quality-of-life outcomes during the statistical mediation analysis**

| **Outcome** | **Complete-case N** | **Stroke, n (%)** | **Controls, n (%)** | **Original sets, n** | **Sets remaining, n (%)** | **Sets retaining both groups, n (%)** | **Control-only sets, n (%)** | **Stroke-only sets, n (%)** | **Completely removed sets, n (%)** | **1 stroke + 3 controls, n (%)** | **1 stroke + 2 controls, n (%)** | **1 stroke + 1 control, n (%)** |
| --- | --- | --- | --- | --- | --- | --- | --- | --- | --- | --- | --- | --- |
| Physical QOL | 535 | 143 (26.7%) | 392 (73.3%) | 149 | 149 (100.0%) | 143 (96.0%) | 6 (4.0%) | 0 (0.0%) | 0 (0.0%) | 100 (69.9%) | 37 (25.9%) | 6 (4.2%) |
| Psychological QOL | 535 | 143 (26.7%) | 392 (73.3%) | 149 | 149 (100.0%) | 143 (96.0%) | 6 (4.0%) | 0 (0.0%) | 0 (0.0%) | 100 (69.9%) | 37 (25.9%) | 6 (4.2%) |
| Social QOL | 532 | 143 (26.9%) | 389 (73.1%) | 149 | 149 (100.0%) | 142 (95.3%) | 6 (4.0%) | 1 (0.7%) | 0 (0.0%) | 99 (69.7%) | 37 (26.1%) | 6 (4.2%) |
| Environmental QOL | 535 | 143 (26.7%) | 392 (73.3%) | 149 | 149 (100.0%) | 143 (96.0%) | 6 (4.0%) | 0 (0.0%) | 0 (0.0%) | 100 (69.9%) | 37 (25.9%) | 6 (4.2%) |
| Overall QOL | 535 | 143 (26.7%) | 392 (73.3%) | 149 | 149 (100.0%) | 143 (96.0%) | 6 (4.0%) | 0 (0.0%) | 0 (0.0%) | 100 (69.9%) | 37 (25.9%) | 6 (4.2%) |
| Overall health | 535 | 143 (26.7%) | 392 (73.3%) | 149 | 149 (100.0%) | 143 (96.0%) | 6 (4.0%) | 0 (0.0%) | 0 (0.0%) | 100 (69.9%) | 37 (25.9%) | 6 (4.2%) |

^Notes: Percentages for the 1:3, 1:2 and 1:1 matched-set configurations were calculated among sets retaining both stroke and control participants^

**Supplementary Table S8: Sensitivity analysis for unmeasured mediator–outcome confounding in the adjusted statistical mediation model for the physical quality of life domain**

| **Outcome** | **Sample size** | **Indirect effect (ACME), 95% CI** | **ACME p-value** | **Direct effect (ADE), 95% CI** | **Total effect, 95% CI** | **Critical rho at ACME = 0** | **R2-star product** | **R2-tilde product** |
| --- | --- | --- | --- | --- | --- | --- | --- | --- |
| Physical QoL | 535 | -0.67 (-1.35 to -0.19) | 0.001 | -5.54 (-8.22 to -2.99) | -6.21 (-8.90 to -3.64) | 0.16 | 0.0256 | 0.0168 |

^Notes: The indirect, direct, and total association estimates, confidence intervals, and p-values were obtained from the primary matched-set bootstrap analysis. The critical ρ and R² sensitivity parameters were obtained separately using a model-based sensitivity analysis and were not based on matched-set bootstrap resampling.^

**Supplementary Table S9:** **Post-hoc exploratory statistical indirect-association analyses between stroke and each of the seven individual WHOQOL-BREF physical-domain items through IDEA scores using separate item-specific models on their five-point Likert scale.**

| **QOL outcome** | **Model** | **Indirect association beta (95% CI)** | **Indirect association raw p** | **Indirect association BH-adjusted p** | **Direct association beta (95% CI)** | **Direct association raw p** | **Direct association BH-adjusted p** | **Total association beta (95% CI)** | **Total association raw p** | **Total association BH-adjusted p** |
| --- | --- | --- | --- | --- | --- | --- | --- | --- | --- | --- |
| Pain interference (Item 3) | Unadjusted | -0.037 (-0.085 to -0.006) | 0.014* | 0.037* | -0.448 (-0.668 to -0.223) | <0.001* | <0.001* | -0.485 (-0.709 to -0.255) | <0.001* | <0.001* |
| Pain interference (Item 3) | Adjusted | -0.051 (-0.113 to -0.009) | 0.004* | 0.028* | -0.401 (-0.640 to -0.152) | 0.005* | 0.018* | -0.452 (-0.700 to -0.197) | <0.001* | <0.001* |
| Medical treatment need (Item 4) | Unadjusted | -0.021 (-0.050 to -0.000) | 0.040* | 0.070 | -0.600 (-0.802 to -0.399) | <0.001* | <0.001* | -0.621 (-0.817 to -0.428) | <0.001* | <0.001* |
| Medical treatment need (Item 4) | Adjusted | -0.027 (-0.064 to 0.002) | 0.082 | 0.096 | -0.297 (-0.504 to -0.090) | 0.009* | 0.020* | -0.324 (-0.530 to -0.120) | 0.004* | 0.009* |
| Energy (Item 10) | Unadjusted | -0.033 (-0.066 to -0.007) | 0.011* | 0.037* | -0.230 (-0.403 to -0.057) | 0.010* | 0.012* | -0.263 (-0.437 to -0.089) | 0.003* | 0.004* |
| Energy (Item 10) | Adjusted | -0.032 (-0.070 to -0.005) | 0.021* | 0.054 | -0.139 (-0.330 to 0.043) | 0.139 | 0.162 | -0.171 (-0.359 to 0.010) | 0.071 | 0.083 |
| Mobility (Item 15) | Unadjusted | -0.028 (-0.064 to -0.004) | 0.016* | 0.037* | -0.342 (-0.501 to -0.188) | <0.001* | <0.001* | -0.371 (-0.527 to -0.214) | <0.001* | <0.001* |
| Mobility (Item 15) | Adjusted | -0.030 (-0.068 to -0.004) | 0.023* | 0.054 | -0.311 (-0.487 to -0.135) | <0.001* | <0.001* | -0.342 (-0.521 to -0.164) | <0.001* | <0.001* |
| Sleep (Item 16) | Unadjusted | -0.001 (-0.017 to 0.015) | 0.921 | 0.921 | -0.101 (-0.231 to 0.023) | 0.121 | 0.121 | -0.102 (-0.234 to 0.025) | 0.123 | 0.123 |
| Sleep (Item 16) | Adjusted | -0.004 (-0.025 to 0.014) | 0.611 | 0.611 | -0.033 (-0.173 to 0.118) | 0.636 | 0.636 | -0.037 (-0.181 to 0.118) | 0.605 | 0.605 |
| Daily activities (Item 17) | Unadjusted | -0.013 (-0.040 to 0.003) | 0.115 | 0.161 | -0.263 (-0.413 to -0.118) | <0.001* | <0.001* | -0.276 (-0.425 to -0.131) | <0.001* | <0.001* |
| Daily activities (Item 17) | Adjusted | -0.023 (-0.060 to 0.003) | 0.077 | 0.096 | -0.187 (-0.340 to -0.034) | 0.014* | 0.020* | -0.210 (-0.373 to -0.050) | 0.007* | 0.012* |
| Work capacity (Item 18) | Unadjusted | -0.010 (-0.030 to 0.004) | 0.164 | 0.191 | -0.231 (-0.374 to -0.098) | <0.001* | <0.001* | -0.242 (-0.385 to -0.106) | <0.001* | <0.001* |
| Work capacity (Item 18) | Adjusted | -0.016 (-0.040 to 0.002) | 0.077 | 0.096 | -0.171 (-0.325 to -0.026) | 0.013* | 0.020* | -0.187 (-0.342 to -0.040) | 0.009* | 0.013* |

**^Notes:^** ^Path a represents the association between stroke status and IDEA cognition score. Path b represents the association between IDEA cognition score and the relevant QOL item, conditional on stroke status and model covariates. The indirect association was calculated as the product of paths a and b. The direct association represents the association between stroke status and the relevant QOL item, conditional on IDEA cognition score and model covariates. The total association represents the association between stroke status and the relevant QOL item, excluding the IDEA cognition score from the outcome model. Items 3 and 4 were reverse-scored so that higher scores consistently indicated better QOL. In the unadjusted analysis, stroke status was modelled as a predictor of the IDEA cognition score (path a), and stroke status and the IDEA cognition score were included as predictors of the relevant QOL item (path b). Adjusted models controlled for age, sex, ethnicity, marital status, education, current employment, hypertension, diabetes, high cholesterol, heart disease, and smoking history. Dependence within the 149 propensity-score-matched sets was addressed by resampling entire matched sets with replacement. Confidence intervals are percentile intervals based on 2,000 matched-set bootstrap samples. The Benjamini–Hochberg correction was applied separately across the seven physical-domain items in the unadjusted and adjusted analyses, and separately for the indirect, direct, and total associations. Outcome-specific complete-case analysis was used.^

**Supplementary Table S10: Participant characteristics by stroke status before and after multiple imputation**

| **Variable** | **Category** | **Before MI, No stroke** | **Before MI, Stroke** | **Before, p** | **After MI, No stroke** | **After MI, Stroke** | **After MI, p** |
| --- | --- | --- | --- | --- | --- | --- | --- |
| IDEA total score | Median [IQR] | 13.00 [11.00–15.00] | 13.00 [10.00–15.00] | 0.004* | 13.00 [11.00–15.00] | 13.00 [10.00–15.00] | 0.004* |
|  | Mean (SD) | 12.55 (2.98) | 11.74 (3.39) | 0.010* | 12.55 (2.98) | 11.74 (3.39) | 0.010* |
| Age, years | Median [IQR] | 70.00 [65.00–74.00] | 70.00 [65.00–74.00] | 0.993 | 70.00 [65.00–74.00] | 70.00 [65.00–74.00] | 0.993 |
|  | Mean (SD) | 70.35 (6.79) | 70.38 (6.90) | 0.956 | 70.35 (6.79) | 70.38 (6.90) | 0.956 |
| Sex | Female | 186 (41.6%) | 62 (41.6%) | 1.000 | 186 (41.6%) | 62 (41.6%) | 1.000 |
|  | Male | 261 (58.4%) | 87 (58.4%) |  | 261 (58.4%) | 87 (58.4%) |  |
| Ethnicity | Malay | 375 (83.9%) | 107 (71.8%) | 0.003* | 375 (83.9%) | 107 (71.8%) | 0.003* |
|  | Chinese | 45 (10.1%) | 23 (15.4%) |  | 45 (10.1%) | 23 (15.4%) |  |
|  | Indian/Other | 27 (6.0%) | 19 (12.8%) |  | 27 (6.0%) | 19 (12.8%) |  |
| Marital status | Not married | 120 (26.8%) | 35 (23.5%) | 0.419 | 120 (26.8%) | 35 (23.5%) | 0.419 |
|  | Married | 327 (73.2%) | 114 (76.5%) |  | 327 (73.2%) | 114 (76.5%) |  |
| Education level | <6 years of education | 111 (24.8%) | 37 (24.8%) | 1.000 | 111 (24.8%) | 37 (24.8%) | 1.000 |
|  | ≥6 years of education | 336 (75.2%) | 112 (75.2%) |  | 336 (75.2%) | 112 (75.2%) |  |
| Current work status | Currently not working | 311 (69.7%) | 119 (79.9%) | 0.017* | 312 (69.8%) | 119 (79.9%) | 0.017* |
|  | Currently working | 135 (30.3%) | 30 (20.1%) |  | 135 (30.2%) | 30 (20.1%) |  |
| Hypertension | Not hypertensive | 165 (38.6%) | 31 (21.1%) | <0.001* | 173 (38.7%) | 31 (21.0%) | <0.001* |
|  | Hypertensive | 262 (61.4%) | 116 (78.9%) |  | 274 (61.3%) | 118 (79.0%) |  |
| Diabetes | Not diabetic | 293 (70.1%) | 74 (50.7%) | <0.001* | 315 (70.4%) | 76 (50.9%) | <0.001* |
|  | Diabetic | 125 (29.9%) | 72 (49.3%) |  | 132 (29.6%) | 73 (49.1%) |  |
| High cholesterol | No high cholesterol | 169 (42.1%) | 31 (21.1%) | <0.001* | 193 (43.1%) | 32 (21.3%) | <0.001* |
|  | High cholesterol | 232 (57.9%) | 116 (78.9%) |  | 254 (56.9%) | 117 (78.7%) |  |
| Heart disease | No heart disease | 404 (90.8%) | 131 (87.9%) | 0.311 | 406 (90.8%) | 131 (87.9%) | 0.311 |
|  | Heart disease | 41 (9.2%) | 18 (12.1%) |  | 41 (9.2%) | 18 (12.1%) |  |
| Smoking | No history of smoking | 307 (68.7%) | 90 (60.4%) | 0.064 | 307 (68.7%) | 90 (60.4%) | 0.064 |
|  | History of smoking | 140 (31.3%) | 59 (39.6%) |  | 140 (31.3%) | 59 (39.6%) |  |

^MI, Multiple imputation^

^Welch’s t-test was used to compare the means of continuous variables between two groups.^

^Mann–Whitney U test was used to compare the distributions or medians of continuous variables that were not normally distributed.^

^Pearson’s chi-square test was used to compare categorical variables when expected cell counts were adequate.^

^Fisher’s exact test was used for categorical variables when expected cell counts were small (when any expected count was <5)^

**Supplementary Table S11.** Statistical indirect association between stroke and physical quality of life through cognitive function following multiple imputation (N = 596)

| **Outcome** | **Model** | **Path a** | **Path a 95% CI** | **Path a p** | **Path b** | **Path b 95% CI** | **Path b p** | **Indirect effect** | **Indirect 95% CI** | **Indirect p** | **Direct effect** | **Direct 95% CI** | **Direct p** | **Total effect** | **Total 95% CI** | **Total p** | **Proportion mediated percent** | **Proportion mediated 95% CI** | **Imputations** |
| --- | --- | --- | --- | --- | --- | --- | --- | --- | --- | --- | --- | --- | --- | --- | --- | --- | --- | --- | --- |
| Physical QOL | Unadjusted | -0.81 | -1.42 to -0.19 | 0.010* | 0.67 | 0.33 to 1.01 | <0.001* | -0.54 | -1.03 to -0.04 | 0.033* | -7.91 | -10.50 to -5.31 | <0.001* | -8.45 | -11.05 to -5.84 | <0.001* | 6.4 | 0.22 to 12.54 | 20 |
| Physical QOL | Adjusted | -0.83 | -1.47 to -0.20 | 0.010* | 0.69 | 0.33 to 1.05 | <0.001* | -0.58 | -1.10 to -0.06 | 0.030* | -5.67 | -8.43 to -2.92 | <0.001* | -6.25 | -9.08 to -3.42 | <0.001* | 9.2 | 0.08 to 18.34 | 20 |

**Notes:** Path a represents the association between stroke status and cognitive function. Path b represents the association between cognitive function and physical QOL, conditional on stroke status. The indirect association is the product of paths a and b. Estimates were pooled across 20 multiply imputed datasets using Rubin's rules. Each imputed dataset was analysed using 2,000 matched-set bootstrap resamples, yielding a total of 40,000 bootstrap estimates. CI, confidence interval; QOL, quality of life.

**Supplementary Table S12. Sociodemographic and clinical characteristics of the matched study sample, overall and stratified by sex (N = 596)**

| **Variable** | **Category** | **Total (n = 596)** | **Female (n = 248)** | **Male (n = 348)** | **P value** |
| --- | --- | --- | --- | --- | --- |
| IDEA cognition score | Mean (SD) | 12.35 (3.10) | 12.25 (3.28) | 12.42 (2.97) | 0.518 |
|  | Median [IQR] | 13.00 [11.00–15.00] | 13.00 [10.00–15.00] | 13.00 [11.00–15.00] | 0.990 |
| Age, years | Mean (SD) | 70.36 (6.81) | 70.01 (7.60) | 70.60 (6.20) | 0.310 |
|  | Median [IQR] | 70.00 [65.00–74.00] | 68.50 [64.00–74.00] | 71.00 [65.00–74.00] | 0.029* |
| Stroke status | No stroke | 447 (75.0%) | 186 (75.0%) | 261 (75.0%) | 1.000 |
|  | Stroke | 149 (25.0%) | 62 (25.0%) | 87 (25.0%) |  |
| Ethnicity | Malay | 482 (80.9%) | 206 (83.1%) | 276 (79.3%) | 0.517 |
|  | Chinese | 68 (11.4%) | 25 (10.1%) | 43 (12.4%) |  |
|  | Indian/Other | 46 (7.7%) | 17 (6.9%) | 29 (8.3%) |  |
| Marital status | Not married | 155 (26.0%) | 99 (39.9%) | 56 (16.1%) | <0.001* |
|  | Married | 441 (74.0%) | 149 (60.1%) | 292 (83.9%) |  |
| Education | < 6 years of education | 148 (24.8%) | 92 (37.1%) | 56 (16.1%) | <0.001* |
|  | ≥6 years of education | 448 (75.2%) | 156 (62.9%) | 292 (83.9%) |  |
| Currently working | No | 430 (72.3%) | 228 (91.9%) | 202 (58.2%) | <0.001* |
|  | Yes | 165 (27.7%) | 20 (8.1%) | 145 (41.8%) |  |
| Hypertension | No | 196 (34.1%) | 67 (27.5%) | 129 (39.1%) | 0.004* |
|  | Yes | 378 (65.9%) | 177 (72.5%) | 201 (60.9%) |  |
| Diabetes | No | 367 (65.1%) | 144 (60.0%) | 223 (68.8%) | 0.030* |
|  | Yes | 197 (34.9%) | 96 (40.0%) | 101 (31.2%) |  |
| High cholesterol | No | 200 (36.5%) | 79 (33.9%) | 121 (38.4%) | 0.279 |
|  | Yes | 348 (63.5%) | 154 (66.1%) | 194 (61.6%) |  |
| Heart disease | No | 535 (90.1%) | 226 (91.5%) | 309 (89.0%) | 0.325 |
|  | Yes | 59 (9.9%) | 21 (8.5%) | 38 (11.0%) |  |
| History of smoking | No | 397 (66.6%) | 247 (99.6%) | 150 (43.1%) | <0.001* |
|  | Yes | 199 (33.4%) | 1 (0.4%) | 198 (56.9%) |  |

^Welch’s t-test was used to compare the means of continuous variables between two groups.^

^Mann–Whitney U test was used to compare the distributions or medians of continuous variables that were not normally distributed.^

^Pearson’s chi-square test was used to compare categorical variables when expected cell counts were adequate.^

^Fisher’s exact test was used for categorical variables when expected cell counts were small (when any expected count was <5)^

^Totals may not equal 596 because of missing data for some variables^.

**Supplementary Table S13: Pairwise correlations among WHOQOL-BREF domains, overall quality of life, and overall health in the matched sample**

| **Variable 1** | **Variable 2** | **Pairwise N** | **r** | **p (raw)** | **p (adjusted)** |
| --- | --- | --- | --- | --- | --- |
| Physical QOL | Psychological QOL | 595 | 0.57 | <0.001* | <0.001* |
| Physical QOL | Social relationships QOL | 591 | 0.27 | <0.001* | <0.001* |
| Physical QOL | Environmental QOL | 595 | 0.42 | <0.001* | <0.001* |
| Physical QOL | Overall QOL | 595 | 0.25 | <0.001* | <0.001* |
| Physical QOL | Overall health | 595 | 0.45 | <0.001* | <0.001* |
| Psychological QOL | Social relationships QOL | 591 | 0.35 | <0.001* | <0.001* |
| Psychological QOL | Environmental QOL | 595 | 0.60 | <0.001* | <0.001* |
| Psychological QOL | Overall QOL | 595 | 0.41 | <0.001* | <0.001* |
| Psychological QOL | Overall health | 595 | 0.40 | <0.001* | <0.001* |
| Social relationships QOL | Environmental QOL | 591 | 0.42 | <0.001* | <0.001* |
| Social relationships QOL | Overall QOL | 591 | 0.18 | <0.001* | <0.001* |
| Social relationships QOL | Overall health | 591 | 0.16 | <0.001* | <0.001* |
| Environmental QOL | Overall QOL | 595 | 0.27 | <0.001* | <0.001* |
| Environmental QOL | Overall health | 595 | 0.24 | <0.001* | <0.001* |
| Overall QOL | Overall health | 596 | 0.32 | <0.001* | <0.001* |

*Note: r = Pearson’s correlation coefficient. Adjusted p-values were calculated using the Holm correction for 15 pairwise comparisons.

An asterisk (*) indicates statistical significance at p < 0.05.

**Supplementary Table S14. Average inter-domain correlations among the four WHOQOL-BREF domains, stratified by stroke status**

| **Group** | **N** | **Number of correlations** | **Average inter-domain correlation (r)** |
| --- | --- | --- | --- |
| Stroke survivors | 149 | 6 | 0.43 |
| Non-stroke participants | 447 | 6 | 0.43 |

**Supplementary Table S15. Simulation-based sample size requirements to achieve 80% power for detecting indirect associations under a 1:3 stroke-to-control ratio**

| Scenario | Path a | Path b | Stroke participants | Control participants | Total sample | Estimated power |
| --- | --- | --- | --- | --- | --- | --- |
| Small–Small | 0.14 | 0.14 | 275 | 825 | 1100 | 83.8% |
| Small–Medium | 0.14 | 0.39 | 175 | 525 | 700 | 85.0% |
| Small–Large | 0.14 | 0.59 | 175 | 525 | 700 | 86.8% |
| Medium–Small | 0.39 | 0.14 | 200 | 600 | 800 | 82.2% |
| Medium–Medium | 0.39 | 0.39 | 50 | 150 | 200 | 99.0% |
| Medium–Large | 0.39 | 0.59 | 25 | 75 | 100 | 90.8% |
| Large–Small | 0.59 | 0.14 | 250 | 750 | 1000 | 83.1% |
| Large–Medium | 0.59 | 0.39 | 50 | 150 | 200 | 97.6% |
| Large–Large | 0.59 | 0.59 | 25 | 75 | 100 | 99.8% |

**Notes.** Path a represents the association between stroke and cognitive function, and path b represents the association between cognitive function and the outcome. Values represent the minimum sample size required to achieve at least 80% power under a planned 1:3 stroke-to-control ratio based on simulation.

**Supplementary Table S15 answers the following question: How many participants would be required in future studies to achieve at least 80% power to detect indirect associations of varying magnitudes using the analytical approach implemented in the present study?**

**Supplementary Table S16. Simulation-based power of the current study sample (149 stroke participants and 447 matched controls; total N = 596) under assumed indirect association scenarios**

| **Scenario** | **Path a** | **Path b** | **Stroke participants** | **Control participants** | **Total sample** | **Estimated power (%)** |
| --- | --- | --- | --- | --- | --- | --- |
| Small–Small | 0.14 | 0.14 | 149 | 447 | 596 | 59.6 |
| Small–Medium | 0.14 | 0.39 | 149 | 447 | 596 | 80.8 |
| Medium–Small | 0.39 | 0.14 | 149 | 447 | 596 | 68.0 |
| Medium–Medium | 0.39 | 0.39 | 149 | 447 | 596 | 100.0 |
| Small–Large | 0.14 | 0.59 | 149 | 447 | 596 | 78.2 |
| Large–Small | 0.59 | 0.14 | 149 | 447 | 596 | 60.0 |
| Medium–Large | 0.39 | 0.59 | 149 | 447 | 596 | 100.0 |
| Large–Medium | 0.59 | 0.39 | 149 | 447 | 596 | 100.0 |
| Large–Large | 0.59 | 0.59 | 149 | 447 | 596 | 100.0 |

**^Notes.^** ^Path a represents the standardised association between stroke and cognitive function, and path b represents the standardised association between cognitive function and the outcome. Small, medium, and large standardized path coefficients (0.14, 0.39, and 0.59, respectively) were based on Fritz and MacKinnon's mediation effect-size scenarios. Estimated power is the probability of detecting a statistically significant indirect association at the observed sample size (149 stroke participants and 447 matched controls; total N = 596).^

Supplementary Table S16 addresses a second, complementary question: With the current sample size (149 stroke participants and 447 matched controls), what level of statistical power would be expected to detect indirect associations of varying magnitudes using the same analytical approach implemented in this study?

1.	Fritz MS, MacKinnon DP. Research Article: Required Sample Size to Detect the Mediated Effect. Psychol Sci. 2007;18(3):233. doi: 10.1111/j.1467-9280.2007.01882.x

2.	Little RJA. A Test of Missing Completely at Random for Multivariate Data with Missing Values. Journal of the American Statistical Association. 1988;83(404):1198–202. doi: 10.1080/01621459.1988.10478722
